# Research for universal health coverage: setting priorities for policy and systems research in Uganda

**DOI:** 10.1080/16549716.2021.1956752

**Published:** 2021-08-17

**Authors:** Freddie Ssengooba, Aloysius Ssennyonjo, Elizeus Rutebemberwa, Timothy Musila, Suzanne Namusoke Kiwanuka, Enid Kemari, Milly Nattimba

**Affiliations:** a Department of Health Policy, Planning and Management, School of Public Health Makerere University Kampala Uganda, Uganda; b Department of Policy and Planning, Ministry of Health, Kampala, Uganda

**Keywords:** Priority setting, health policy and systems research, multi-voting, universal health coverage, Uganda

## Abstract

**Background:**

There is international consensus on the need for countries to work towards achieving universal health coverage (UHC) whereby the population is given access to all appropriate promotive, preventive, curative and rehabilitative services at affordable cost. The World Health Organisation (2013) urges all countries to undertake research to customise UHC within national development agendas.

**Objective:**

To describe the process used to prioritise UHC within the health systems research and development agenda in Uganda.

**Methods:**

Two national consultative workshops were convened in May and August 2015 to develop a UHC research agenda in Uganda. The participants included multisector representatives from local, national, and international organisations. A participatory approach with structured deliberations and multi-voting techniques was used. Stakeholders’ views were analysed thematically according to health systems building blocks, and multi-voting was used to assign priorities across themes and sub-themes. The priorities were further validated and disseminated at national health sector meetings.

**Results:**

Of the 80 invited stakeholders, 57 (71.3%) attended. The expressed priorities were: 1) health workforce; 2) governance; 3) financing; 4) service delivery, and 5) community health. The participants also recommended crosscutting research themes to address the social determinants of health, multisectoral collaboration, and health system resilience to protect against external shocks and disease epidemics.

**Conclusion:**

Discussions that capture the diverse perspectives of stakeholders provide a way of exploring UHC within health policy and systems development. In Uganda, attention should be paid to the principal challenges of mobilising financial and technical capabilities for research and strengthening the link between evidence generation and policy actions to achieve UHC.

## Introduction

In September 2015, there was a global agreement on the need for countries to make deliberate efforts to achieve universal health coverage (UHC), defined as ‘securing access to all appropriate promotive, preventive, curative and rehabilitative services at an affordable cost’ [1–3]. UHC is a powerful instrument for attaining better health and wellbeing and fostering human development [4]. However, there is no prescriptive path to UHC, and every country has to carve out its own course. UHC requires policy actions to be taken within a complex and increasingly multisectoral arena. Legitimate role-bearers include the Ministries of Health, Finance, Education, Labour, Housing and Social Development [5] as well as state actors (i.e. development partners, and representatives from private sector and civil society organisations and the media) [6]. This requires whole-of-government and whole-of-society approaches [7]. Moreover, the need for functional health systems to address the health needs of a country’s population while protecting the people from financial hardships and ensuring equity in access to the needed healthcare services has been emphasised as a prerequisite for moving towards UHC [8]. For example, the United Nations General Assembly (2019) at its 74^th^ session in New York underscored the need to incentivise public health research to support UHC and maintain the core principles of safety, affordability, effectiveness, efficiency, equity and shared responsibility for all member states [9].

In Uganda, the Ministry of Health committed to achieving UHC in the Health Sector Development Plan (HSDP) 2015/16-2019/2020. The aim was ‘to accelerate movement towards universal health coverage with essential health and related services needed for the promotion of a healthy and productive life’ [10,11].

Over the last decade, there has been increased interest in health policy and systems research (HPSR) [12]. The interest in HPSR is built on the understanding that each country needs to customise the UHC and other policy agendas and pathways guided by research evidence and learning systems for progressive improvements [13,14]. HPSR is needed to inform the design and implementation of health sector reforms [15,16]. According to the World Health Report 2013 [1], research can answer concerns and questions about how UHC can be advanced, thereby providing solutions to improve human health, wellbeing and development. The HPSR is also vital for monitoring progress towards UHC. Research is critical for health systems development and evidence-informed decision-making [12]. Nevertheless, there is a shortage of information on the national priority research agenda to advance UHC. Countries are urged to undertake research as they work towards achieving UHC [17].

The World Health Report [1] states that ‘*to make the best use of limited resources, systems are needed to develop national research agendas, to raise funds, to strengthen research capacity, and to make appropriate and effective use of research findings*’ (pg. ix). The same report identifies four functions to address health systems research, namely a) defining research questions and priorities; b) raising funds and developing research staff capacity and infrastructure; c) establishing norms and standards for research practice; and d) translating research findings to guide policy. The report further states that priority should be given to the health research system at the national level that includes the people (individuals, groups or organisations), institutions and activities working primarily to generate evidence and information to improve population health [18]. Establishing Uganda’s policy and systems research agenda was intended to kick-start the cascade of these interlinked objectives.

This paper focuses on the first function above. It describes the process used to prioritise UHC within the health systems research and development agenda in Uganda. This work provides valuable lessons for other countries in setting their research priorities for UHC. The COVID-19 pandemic has increased the need for countries to undertake nationally relevant research to help manage the pandemic’s operational aspects of healthcare systems and mitigate the pandemic’s impacts on national policy agendas, including UHC [19].

## Methods

### Study design

This was a structured deliberative process employing nominal group technique with multi-voting [16,17] involving major stakeholders in discussions about research priorities and policy actions to achieve UHC targets in Uganda. To develop a UHC research agenda in Uganda, two national stakeholder workshops were convened in May and August 2015. The first meeting was held alongside the national consultative meeting about the new HSDP, which was later launched at the end of 2015 [10]. This tag-on workshop’s main objective was to build consensus among stakeholders regarding the priority areas for research to support the UHC agenda during the HSDP period. This workshop session on the research priority for UHC was one of the many workshop sessions organised in 2015 by the Ministry of Health and the Supporting Policy Engagement for Evidence-based Decisions (SPEED) for UHC Project. The session aimed at allowing the national stakeholders to get acquainted with the proposed HSDP and provide inputs before the latter’s launch in the same year. A follow-up workshop was held in late 2015 to validate the findings from the first workshop and build further consensus among additional stakeholders on the priority areas for research on UHC. The second deliberative workshop was also aimed at increasing the range and perspectives of stakeholders not well represented at the first workshop. However, to avoid biasing the views of the second workshop, the findings from the first meeting were not shared. Instead, the entire process of structured deliberation was repeated with the new group. All the authors were part of the organising team for both meetings. FS, AS, ER, SNK and TM focused mainly on the technical aspects of the meetings. AS, EK and MN oversaw the logistical and administrative processes of mobilising participants and documenting deliberations. In each meeting, an overview of UHC dimensions [3] was provided by FS at the start.TM provided the MOH perspective. The sessions were audio-recorded after obtaining approval from the participants. These recordings were transcribed verbatim to ensure that people’s views were well contextualised in the reports. To disseminate and further validate the outcomes, FS and SNK shared the research priorities at the national UHC symposium and the Annual Health Sector Performance Review meeting of the health sector for the same year.

### Data collection and analysis approach

As noted above, during each workshop, a participatory approach known as nominal group technique with multi-voting [20,21] was used to solicit stakeholders’ views based on their experiences. The deliberative and interactive process approach combined individual exercises, group discussions, and plenary deliberations for consensus-building. The meetings lasted one day and were structured as presented below.

#### Brainstorming

The participants were requested to suggest key issues that they personally considered important for research to support UHC progress in Uganda. A 15-minute plenary presentation about UHC and the national aspirations preceded this step. This step was guided by two questions and the unique institutional perspectives/role a participant represented in advancing UHC goals and processes. The questions were framed as priority solutions to scale up or problems to solve. These were stated as: 1) What 2–3 solutions need to be prioritised for scaling up if the UHC vision is to be realised in the next 5–10 years? 2) What 2–3 priority problems need solving to accelerate the attainment of the UHC vision in the next 5–10 years? During this stage, three sticky notes were given to each participant, and each person was requested to write one idea per sticky note. The stickers were categorised along the health systems building blocks as thematic areas [1]. The notes which did not fall under any of the above thematic areas were classified under ‘others’.

#### Categorising emerging sub-themes

Six small groups of 4–5 people each were formed from among the meeting participants. Each group had an opportunity to walk around and examine issues posted on each thematic poster (building blocks such as workforce). As the groups rotated around, the topics posted on each thematic poster were reviewed, discussed and categorised. As a guide for this step, the groups were asked to stand at one of the seven stations around the room, representing a health system building block. They were guided by the authors to examine and categorise the issues on the Post-It notes into sub-themes relevant to that building block and convert these into researchable questions. The first group at a thematic station worked to develop sub-themes from the issues posted at their station. A rapporteur for each station was assigned and remained at the same poster to introduce to the incoming group the issues from the prior group(s) and record additional contributions on the theme and sub-themes. All seven groups had the opportunity to visit all the stations in a merry-go-round fashion. After the discursive walk-arounds, each station’s rapporteur presented to the plenary a summary of the emerging issues for each building block and its sub-themes. All the participants were invited to discuss and refine the sub-themes and emerging research questions. Given the issues posted on the thematic poster labelled as ‘Others’, the plenary discussions observed these related to other determinants of health and were renamed ‘Multisectoral aspects of health’. Across the seven themes, 27 sub-categories were developed at this stage.

#### Multi-voting to assign priority

For this step, the participants were each given eight votes in the form of adhesive sticky notes to cast across all the sub-themes arising from the prior exercise. The participants were guided to vote according to the interests arising from the mandates or constituency they represented at the meeting. For example, the participants representing hospitals would look out for sub-themes likely to affect service provision in the context of UHC aspirations. The participants were told that the prioritisation was informed by the fact that there were not enough resources to address all the research issues as generated from the prior session. They were informed that voting was a way to arrive at and signal priority research topics or themes to the government, research institutions and research funders. All were invited to vote by placing one or more of their adhesive sticky notes next to their perceived priority research sub-themes until they used all the eight sticky notes assigned to them.

#### Deliberations about themes and sub-themes

The authors counted the votes for each sub-theme, and the results were presented to the meeting plenary. The top five sub-themes were further discussed. Refinement and appeals for some popular items outside of the top five were made to the plenary to consider. For example, overlapping sub-themes were merged, and their votes were summed up. Five groups, each with 5–6 people, were formed using volunteering and matching themes with relevant institutional roles, interests or expertise needed to address the top five research sub-themes. Each group was asked to develop specific research questions and harmonise, where necessary, the existing overlaps from the previous steps. Each group briefly presented the key research questions to the plenary session, and this was followed by brief deliberations as the final activity.

## Results

### Participants and overview of results

**Figure 1.**
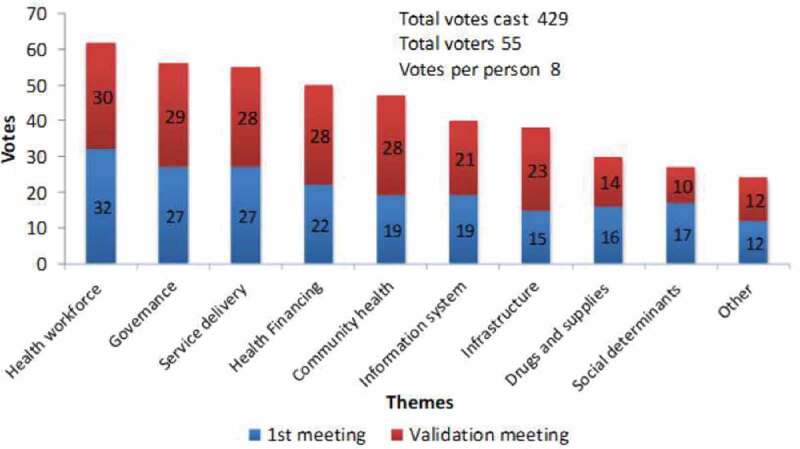
Ranking of priority research themes

The two consultative meetings were attended by 57 participants, who cast a total of 429 votes. The results of votes cast for the priority theme are presented in Figure 1.

The participants included district health managers, hospital directors, health service researchers, policymakers, research funders and representatives of local and international organisations. These were chosen basing on their expert knowledge and were drawn from different professional and organisational backgrounds to harness the multidisciplinary and diverse perspectives needed to play active roles in the UHC agenda in Uganda. For the analysis, the views and contributions from the two workshops were combined to generate the overall research priorities and thematic descriptive texts about the contextual issues, problems or solutions related to the research priorities. Table 1 below summarises participation by individuals and organisations.

**Table 1.** Participation at deliberative workshops

Activity	Individuals* attending (% of invited to attended).	Institutions attending (% of invited to attended).
**UHC Research agenda -First Workshop (26 May 2015)**	27 (39)	8 (32)
**UHC Research Agenda-Validation Workshop (13 August 2015)**	30 (67)	14 (64)

*There was possibility that some people especially SPEED team members attended both meetings.

A total of 10 broad research problems and questions from the last step were further grouped into six themes according to health systems’ building blocks. The participants felt that the research agenda for UHC should prioritise research questions regarding the different themes in the above order (Figure 1). The next subsection provides an overview of the main issues under each theme.

### Results per theme

#### Health workforce

The theme of the health workforce was ranked highest. The participants framed the issues as either problems or solutions. The problems identified included: 1) Inadequate numbers and skills among the health workers; 2) Low productivity of the human resources for health (HRH); 3) Inadequate performance management for frontline managers; and 4) Insufficient remuneration and facilitation for HRH. Some participants suggested solutions. These were mirror images of the problems and included capacity development, task shifting, improving regulatory bodies’ oversight capacity, and enhancing HRH motivation and productivity.

##### Workforce numbers and skills

Several gaps in knowledge were highlighted under this sub-theme. For example, the participants pointed out the gap between current numbers and projections to estimate the future need for health workers. The participants recommended research to determine and address current and future health workforce gaps and community engagement in health production. There was a call for research to examine how community health workers can be expanded and facilitated to improve community health services geared towards prevention and health promotion.

‘We need to see how to take advantage of the very wide workforce that we have in the communities and households. When we talk of the workforce, we always talk about (clinical) health workers but forget the actual health workers – the household and community health workers. So, the question is: How do we structure the participation of these groups in the health system to reduce the burden of unnecessary deployment of (professional) health workers on issues that communities can handle?’

‘What we are experiencing in the hospitals as of now are old staff establishment standards which prescribe one consultant or even one medical officer. That structure cannot fit the (current) needs. So, what we should be thinking about is research to guide the staffing standards.’

Research on the models, quality and standards for training were also raised. Exploring how to make in-service training more accessible and affordable was considered vital to motivate those health workers already serving. The participants wanted to explore in-service training models that minimise disruption to service delivery due to staff going away for a long time. Likewise, short-term training models – mostly workshops – were also questioned regarding their impact on HRH performance. Many participants expressed concerns about better regulation of the medical training market. At the time, the prevailing context was the numerous training schools that had started but did not have adequate access to hospitals for their trainees’ practical training and skills development. Many new health programmes used short-term workshops as a model for building up skills and scaling up programmes like vaccinations, malaria and HIV care. Research to explore quality gaps, costs and ways to improve workforce performance was among the issues prioritised.

‘The quality of cadres we are channelling through the training institutions is unsatisfactory. We have many private institutions training cadres under the allied health workers category – laboratory technicians, laboratory assistants etc. I had an experience when someone who wanted a laboratory technician job came for an interview but could not write the word “technician”, yet she had trained for two years. Therefore, that concerns the quality of training. More so, who are the people training these cadres?’

##### Performance management

Regarding performance management, HRH motivation and productivity, research was recommended to explore the drivers of motivation, develop adequate incentive packages, and promote customer care skills among the workforce. Questions about the contribution of task shifting to increasing the workforce pool available to handle non-sophisticated services were recommended. For example, one participant inquired: ‘Can we have non-doctors do simple surgical services?’ The discussants also posed questions about how communities could be made producers of health to reduce the unnecessary deployment of professional health workers at the community level.

Disparities in staffing were noted among different geographies (districts and rural-urban divide). Also, recruitment at the district level was reported to be biased towards ‘sons and daughters of the soil’ and not done according to merit-based criteria. Thus, studies were proposed to investigate appropriate staffing standards aligned with the workloads at facilities and decentralised workforce management systems. Research into innovative solutions to broaden the finances for workforce remuneration was also suggested.

#### Governance

##### Regulation of the private sector

The theme of governance identified several problems that required evidence generation and evidence-driven regulatory actions. The regulatory institutions’ capacity and function were also seen as weak and less tuned to the new realities of private health provision. For example, group discussions expressed the multitude of non-governmental organisations (NGOs) that have come to play major roles in programming and policy implementation. The participants noted an enormous expansion of small and medium-sized private entrepreneurs who provided services of varying scale, quality and prices at the community level. The discussants observed that regulatory bodies remained organised around professional groups, regulating and licensing members and enforcing their ethical behaviours. They were less involved in setting standards and rules for the industry. Accreditation of provider organisations and the regulation of prices and quality processes were perceived as largely absent.

‘What capacity do regulatory bodies have to perform their regulatory functions in terms of clarifying policy objectives or clarifying their roles and responsibilities? In terms of human resources, how sufficient are the numbers of regulatory staff? What competencies do they actually have to take on the governance role? Do they have the financing to play this role? Does their financing compromise their roles since funds (sometimes) come from the regulated entities?’

The participants noted that the private sector was a major service provider and lever for attaining UHC goals. Still, data about this sector’s contributions was largely lacking. As such, their contribution to the UHC objective remained unclear for planning purposes. The research questions generated by the discussants centred on finding appropriate, effective and sustainable ways to regulate the private sector – ‘*at a minimum, test the means for tracking service utilisation, prices and quality of services*’.

##### Stewardship for implementation

Cultivating a common vision and strengthening coordination among many stakeholders at the national level were identified as a problem that undercuts the implementation of programmes and policies. Leadership and management capacity was identified as a solution that needs to be cascaded at all policy and implementation levels. To improve the knowledge gaps for stewardship, the participants generated a long list of research gaps. These included negotiating priorities at the community, district and national levels and searching for alternative means of curbing corruption and misappropriation of resources. Other issues included ways to reduce absenteeism and boost the productivity of health workers, how to inculcate entrepreneurial skills and how to empower communities to actively voice their demand for quality services.

*‘Support supervision is one of the key issues that we have considered to reduce staff absenteeism. We all know that some of the health workers are absent from their facilities for a full week. […] That is because there is weak support supervision – they (health workers) know that no one will check on their being at work.’*

#### Service delivery

From the discussions, it was established that service delivery in the country was generally poor in terms of volume and quality. This was attributed to limited engagement of non-state actors, inequitable distribution of the workforce, and sub-optimal functionality of health facilities. Improvements in health outcomes were slow and inequitable. Specific proposals for research included: 1) Improving distribution and functionality of health infrastructure; 2) Monitoring demand and client satisfaction; 3) (Re)designing the benefit package – especially in order to strengthen preventive and promotive services; 4) Introducing performance management systems such as results-based financing; and 5) Strengthening the referral system – especially regarding how to address congestion in tertiary care hospitals.

*‘How can we improve equity in the distribution of health facilities? There is an inequitable distribution of health facilities in the country, and we need to investigate why this is the case. If you count the number of facilities in one region of about seven districts, it is equal to the total facilities in one district in another region. Let me talk of Kamwenge district that has ten facilities while the whole of the Karamoja region has only ten facilities.’*

*‘How can we strengthen the referral systems? Mulago (National Referral Hospital) gets congested, yet we have many lower-level facilities around town underutilised. So, we need to investigate that.’*

A discussion about the design of the benefits package (BP) generated much debate. Research was recommended to determine the causes of low levels and disparities in the coverage of health services and the reasons for the failure of earlier UHC-like agendas. These include drawing lessons from prior efforts at expanding coverage such as ‘*health for all by the year 2000’*. There was general agreement that the benefits package needed to be expanded to include more advanced care but in a manner that is feasible to finance. The participants advocated for pilot studies to assess alternative designs for the benefits package. The participants proposed studies to address the cost drivers for medicines and regulatory mechanisms to curb the supply of fake drugs on the market. Insufficient medicines and supplies were a recurrent factor for the discussions about the quality of health services.

#### Financing

The participants framed health financing problems as arising from 1) high costs of services to the national budget and to communities; 2) inadequate system readiness for health insurance and results-based financing approaches; 3) high dependence of health programmes on external aid; and 4) insufficient domestic financing for health programmes.

The adoption of high-cost interventions like new vaccines for child survival, diagnostics and treatment options for diseases like malaria and HIV were framed as problematic to sustain in the context of a rapidly growing population. The financial burden on the economy arising from expanding health coverage, population size and higher costs of medicines, and wage costs for health workers was viewed as incompatible with UHC’s success. The potential to slow down the UHC coverage agenda seemed inevitable if the cost escalation trends were not controlled. At the time of this study, government-employed health workers had successfully pushed for a 30% wage increase without overall growth in health sector budgets [10,11].

The participants strongly voted for research to address knowledge gaps about health insurance. At the time of this study, social health insurance was top on Uganda's policy agenda, attracting extensive media coverage and resulting in technical consultative meetings. Deliberations at the meetings exhibited consensus on the need to prepare for the insurance and its implications for access to healthcare. The research gaps were identified for national health insurance that was being considered for initiation in the country. These related mostly to health providers’ readiness and the institutional arrangements for purchasing health services. Other concerns related to mitigating known problems like corruption, frivolous utilisation of services and payment delays. *‘This (health insurance) is a subject that raises very many research questions. So, the first question is about the dynamics of insurance rollout. The second is the public facilities and informal sector’s role in the introduction and implementation of the health insurance scheme. The next question is: What is the preparedness of these sectors before the adoption of the scheme?’*

Research priorities here included the costing of services to help set prices and research to monitor the intersection between billing and the distortions in the health information system. Pilots and studies about results-based financing (RBF) were considered as opportunities to help prepare the health system for national health insurance. Nonetheless, RBF schemes’ customisation to the Ugandan health system was considered a priority research activity – partly because these pilots were perceived, at the time, as propagating designs to bypass public institutions in the health sector.

Despite dilemmas in its management, donor aid was seen as vital to the prospects for attaining UHC objectives. The proliferation of funding streams by the global health initiatives was framed both as an opportunity and a challenge to the UHC agenda. Research to explore ways to pool funds across external aid providers and domestic funds (basket funding) was proposed:

*‘There is a question that is always asked about tracking the investments in health: Where does so much money that comes from donors go? We know districts have benefited from this (donor) support, but they are not doing better. Where does that money go? Are these investments really translated into actual outcomes? Can this money be pooled into a common basket to support all programmes?’*

#### Community health

The problem here was framed as low community empowerment, weak governance and accountability for health promotion programmes. The participants concurred on the need to strengthen the evidence about community health systems.

The participants highlighted the need to popularise the UHC agenda at the sub-national level and to create a common understanding of what UHC means to ordinary Ugandans. They thus called for research solutions for 1) community production of health; 2) improvement of community health literacy; 3) better implementation of community-based health programmes; and 4) effective advocacy for health improvements within the communities. It was proposed that further study be undertaken on knowledge gaps in citizen participation in healthcare planning and delivery. Special attention was also drawn towards poor urban communities, mobile and refugee populations, border communities, and institutionalised communities such as uniformed groups and schools. It was proposed that research be carried out to address how to target and reach these special communities for the UHC agenda. “*We know that there are some resources in the community, but how do we harness those resources for better health is the question. So, we, as researchers, need to find answers to this question. What are the characteristics or attributes of an empowered community? … .*

*The other question is: How functional are the current community health systems? [… .]; what influences the functionality of community health systems? […], Researchers should go, investigate and get answers to such questions.”*

#### Other issues

Other issues raised included infrastructure, information systems and social determinants of health. The participants proposed that research prioritise understanding the socio-determinants of health and leveraging multisectoral investments for health improvements. Research about the tools, methods and approaches required to motivate or enable multisectoral collaboration in health was encouraged. Under this theme, a study was proposed to address the rapid population growth – especially scaling up family planning services to reduce population growth and slow down the demand for and costs of healthcare services.

Gaps were explored in accessing and utilising the health information systems (data collection, processing, outputs and their use in decision-making). Although some progress was acknowledged, the adoption of information technology (IT) was considered to be less adequate. Several questions were raised regarding misunderstanding of the UHC agenda and the need to customise UHC goals to Uganda. A consensus from the meeting was that there was need to expand the efforts to engage different stakeholders, harmonise the knowledge disparities and generate evidence about the factors that could promote or hinder the implementation of UHC programmes in Uganda.

## Discussion

There is a global and country commitment to accelerate UHC progress [10]. Research is also expected to play a critical role in supporting the sustainable development agenda [12]. This paper outlines the process and outcomes of a priority-setting exercise for UHC research in Uganda. The structured deliberation methods show the feasibility of generating a suitable priority research agenda that can guide national research and development investments for UHC [22]. As demonstrated in this work, if well mobilised and their deliberations well facilitated, stakeholders can provide a rich set of research ideas that are pertinent to the operational challenges to the success of the UHC agenda. Prioritising research themes and elaborating research questions were needed to ensure that efforts were directed at the topics that stakeholders felt were most urgent or important. In our case, five areas were found to be of paramount importance for UHC in Uganda: workforce for health, governance, service delivery, health financing and community health. Specific sub-themes under each thematic area were elaborated and justified. Other areas of interest, such as medicines and supplies, information systems, infrastructure, social determinants of health, were also identified as vital despite being outside the top five priorities. Crosscutting issues recommended included awareness creation, stakeholder engagement in decision-making and customising the UHC concept to Uganda. For example, research to enhance understanding of how intersectoral policies and actions can be harnessed to improve health and advance development was picked up as a crosscutting issue. As this paper goes to press, the government has elaborated a health sector development plan and UHC roadmap grounded in multisectoral collaboration to address the broader determinants of health.

Overall, attempts were made to customise UHC research to Uganda by specifying the research topics across the key priority areas. These areas are recommended to researchers interested in supporting UHC efforts at the country level. However, there are important factors to be taken into consideration. First, building the capacity to conduct the research. This rests on establishing and maintaining an effective health research system [23].

However, studies reveal that national health research systems are inadequately developed in Africa [24]. There is a need to commit more efforts beyond generating research priorities. Mobilising funds, building capacity for research for people and institutions, and strengthening the link to decision-making remain important challenges. The capacity to conduct health policy and systems research in developing countries remains particularly low [12]. Most of the policy and systems research is not domestically guided or funded and often reflects the priorities of the external funding agency [17].

Institutions like universities, think tanks and policy research experts can play a major role in plugging the gap and becoming major domestic actors in policy developments like UHC and SDG agendas. Agencies like the European Union are among a few other pioneers in establishing grants and programmes, such as the Supporting Public Health Institutes Programme, in developing countries to improve their capacity for health policy analysis and influence [25]. Programmes like the Supporting Policy Engagement for Evidence-based Decisions (SPEED) for UHC in Uganda [26] are among many global projects that benefited from this European Union support.

Ensuring that the research is policy-relevant is vital. Awareness of the evidence and involving decision-makers in the research process is paramount for policy and systems research [12,14]. There is a need to embed research in the discourse for policymaking and practice management [14]. From our experience, the priority-setting exercise was both an awareness activity and a process drawing upon the tactical knowledge of policy problems and solutions the practitioners acquire through their day-to-day experiences.

### Study strengths and limitations

The participatory, deliberative nature of the approach was its main strength. In this case, the engagement of stakeholders – practitioners of policy and systems management in Uganda – was resourceful in generating pertinent policy problems at the core of UHC development in Uganda. To be successful, this methodology rests on a liberal approach to stakeholder identification from all vital perspectives, institutional roles and well-structured deliberations, coupled with expanding the awareness of core concepts like UHC [21]. We successfully used the multi-voting approach to arrive at UHC policy and systems research priorities. The stakeholders appreciated the simplicity and deliberative nature of the approach. Programmes like the SPEED Project continued to be guided by these priorities. They informed their main research products and themes of the policy symposia [27,28].

Limitations of our approach to developing the research agenda are mainly two-fold. There were variable representativeness and experiences of stakeholders and there was a wide range of challenges that required research input. The coverage of the topics and their prioritisation are bound to change if a different mix of stakeholders are invited to participate in the processes we outlined – especially stakeholders outside the health sector. We attempted to mitigate this limitation by holding two separate meetings to broaden the participation. Validation of the priority outcomes at various policy fora enabled broader dissemination of the research priorities, especially within the health sector.

## Conclusion

Realistically, many more research questions can be asked than answered to support the design and implementation of complex programmes like UHC at the country level. It is thus critical and logical to set priorities for policy and system-relevant research. Furthermore, the priorities demonstrate a need to customise research to the national policy discourse and feasible problems and solutions for health systems development.

This research agenda for UHC needs complementary capabilities to be developed along the research-practice nexus – from identifying key research questions through financing to generating the capacity to turn research findings into practical policy and practice applications.

Finally, the research priorities should be seen as dynamic, adaptable and responsive to the health systems’ need to achieve UHC. New challenges emerge, and the priorities need to be refreshed to fit the domestic needs for evidence generation and decision-making. The participatory methodology outlined here can help regular adjustments of the national research agenda for UHC.

## Data Availability

The reports on the meeting are readily available.

## Paper context

This paper demonstrates the application and utility of a participatory process in identifying national priority research questions for UHC and related systems developments. It argues that UHC goals should be adapted to local uncertainties about affordable financing and system readiness to implement the plan. The approach can be applied to different health system levels to generate corresponding research priorities and inform actions for evidence generation and decision-making. The advent of the COVID-19 pandemic has heightened the demand for contextually relevant research. The approach can be applied to research priority-setting for such emergent policy issues.
